# Phosphodiesterase Type 5 Inhibitors and Risk of Malignant Melanoma: Matched Cohort Study Using Primary Care Data from the UK Clinical Practice Research Datalink

**DOI:** 10.1371/journal.pmed.1002037

**Published:** 2016-06-14

**Authors:** Anthony Matthews, Sinéad M. Langan, Ian J. Douglas, Liam Smeeth, Krishnan Bhaskaran

**Affiliations:** Department of Non-Communicable Disease Epidemiology, London School of Hygiene & Tropical Medicine, London, United Kingdom; Harvard Medical School, UNITED STATES

## Abstract

**Background:**

Laboratory evidence suggests that reduced phosphodiesterase type 5 (PDE5) expression increases the invasiveness of melanoma cells; hence, pharmacological inhibition of PDE5 could affect melanoma risk. Two major epidemiological studies have investigated this and come to differing conclusions. We therefore aimed to investigate whether PDE5 inhibitor use is associated with an increased risk of malignant melanoma, and whether any increase in risk is likely to represent a causal relationship.

**Methods and Findings:**

We conducted a matched cohort study using primary care data from the UK Clinical Practice Research Datalink. All men initiating a PDE5 inhibitor and with no prior cancer diagnosis were identified and matched on age, diabetes status, and general practice to up to four unexposed controls. Ever use of a PDE5 inhibitor and time-updated cumulative number of PDE5 inhibitor prescriptions were investigated as exposures, and the primary outcome was malignant melanoma. Basal cell carcinoma, solar keratosis, and colorectal cancer were investigated as negative control outcomes to exclude bias. Hazard ratios (HRs) were estimated from Cox models stratified by matched set and adjusted for potential confounders.

145,104 men with ≥1 PDE5 inhibitor prescription, and 560,933 unexposed matched controls were included. In total, 1,315 incident malignant melanoma diagnoses were observed during 3.44 million person-years of follow-up (mean 4.9 y per person). After adjusting for potential confounders, there was weak evidence of a small positive association between PDE5 inhibitor use and melanoma risk (HR = 1.14, 95% CI 1.01–1.29, *p =* 0.04). A similar increase in risk was seen for the two negative control outcomes related to sun exposure (HR = 1.15, 95% CI 1.11–1.19, *p <* 0.001, for basal cell carcinoma; HR = 1.21, 95% CI 1.17–1.25, *p <* 0.001, for solar keratosis), but there was no increased risk for colorectal cancer (HR = 0.91, 95% CI 0.85–0.98, *p =* 0.01). There was no evidence that risk increased with number of prescriptions received (*p*-trend = 0.83). In a post hoc analysis, there was strong evidence that solar keratosis was associated with future PDE5 inhibitor use (odds ratio = 1.28, 95% CI 1.23–1.34, *p <* 0.001), suggesting that men with higher sun exposure were more likely to become PDE5 inhibitor users. However, a limitation of our study was that we did not have individual-level data on sun exposure, so we could not directly control for this in the primary analysis.

**Conclusions:**

Our results were not consistent with PDE5 inhibitors being causally associated with melanoma risk, and strongly suggest that observed risk increases are driven by greater sun exposure among patients exposed to a PDE5 inhibitor.

## Abstract 2

In an analysis of a large primary-care dataset from the UK, Krishnan Bhaskaran and co-workers report that men who were prescribed phosphodiesterase type 5 inhibitors, such as sildenafil, were unlikely to have a consequent increase in their risk of melanoma.

## Author Summary

**Why Was This Study Done?:**

- An earlier study using US data suggested that users of PDE5 inhibitors, which are drugs widely used for erectile dysfunction, may be at nearly double the risk of developing a type of skin cancer, malignant melanoma; however a subsequent study in Sweden failed to replicate this finding, resulting in considerable uncertainty over the relationship.
- The present study was done to clarify whether PDE5 inhibitors may affect the risk of malignant melanoma.

**What Did the Researchers Do and Find?:**

- Using a large UK-based primary care database, we identified 145,104 men who were prescribed a PDE5 inhibitor and 560,933 matched controls with similar characteristics but no exposure to PDE5 inhibitors; we then compared the subsequent risk of malignant melanoma in these two groups, adjusting for other potentially important factors.
- We observed a small association between the use of PDE5 inhibitors and malignant melanoma (hazard ratio = 1.14, 95% CI 1.01–1.29, *p =* 0.04), but we found evidence of a similar association between the use of PDE5 inhibitors and both basal cell carcinoma and solar keratosis, which are both related to sun exposure and were not hypothesised to be associated with PDE5 inhibitor use.
- We also found strong evidence to suggest that men with a history of solar keratosis, a marker of high sun exposure, were more likely to become PDE5 inhibitor users.

**What Do These Findings Mean?:**

- Our findings were not consistent with PDE5 inhibitors causing a substantial increase in the risk of malignant melanoma.
- It is likely that the small observed increase in the risk of malignant melanoma among PDE5 inhibitor users is explained by higher sun exposure among PDE5 inhibitor users; this is strongly suggested by the increased risk of other diseases related to sun exposure among PDE5 inhibitor users and by the strong association between solar keratosis and subsequent PDE5 inhibitor use, which implies that men with high sun exposure were more likely to become PDE5 inhibitor users.

## Introduction

The phosphodiesterase type 5 (PDE5) inhibitors sildenafil, tadalafil, and vardenafil are principally used in the treatment of erectile dysfunction (ED) [1]. Laboratory evidence suggests that reduced PDE5 expression triggered by BRAF activation increases the invasiveness and metastatic potential of melanoma cells [2]; hence, pharmacological inhibition of PDE5 might have an unintended effect on melanoma risk. In addition, PDE5 inhibitors appear to promote melanin synthesis [3], which in turn can significantly facilitate the development of melanoma [4]. These laboratory observations led to two major epidemiological studies of the effect of PDE5 inhibitor use on melanoma risk, but these came to different conclusions: Li et al. initially reported a near doubling of the hazard of melanoma among sildenafil users within a US-based cohort of health professionals, using self-reported exposure and outcome data (HR = 1.84, 95% CI 1.04–3.22) [5]. However, a subsequent study using Swedish registry data did not support such a large effect size and, despite some overlap in the confidence intervals, suggested a much more modest association between PDE5 inhibitors and melanoma (HR = 1.21, 95% CI 1.08–1.36); the authors expressed doubts over whether even this smaller observed association was causal, as it did not meet several of Hill’s causality criteria [6].

Any increase in malignant melanoma risk caused by PDE5 inhibitor use would have serious public health implications: 5%–20% of men are affected by ED, and PDE5 inhibitors are an effective treatment [7,8]. Furthermore, patents for sildenafil and other drugs have expired or are soon to expire in various countries, leading to the availability of less costly generic versions and the potential for considerably inflated demand in the near future.

Given the importance of the question, and continuing uncertainty over a causal link, we aimed to examine the association between PDE5 inhibitors used for ED and the risk of incident melanoma in a large cohort of men using data from UK primary care, and to assess the causality of any observed increase in risk.

## Methods

### Study Design and Data Source

We carried out a matched cohort study using prospectively collected data from the UK Clinical Practice Research Datalink (CPRD), a database containing anonymised primary care data from general practitioners (GPs) who use the Vision IT system and have agreed at the practice level to participate [9]. The UK has a publicly funded healthcare system that is financed through general taxation and is free at the point of use to UK residents; GPs play a key role as they are responsible for primary healthcare and specialist referrals. Patients are affiliated to a practice, which centralises the medical information from their GP as well as information reported back from specialist referrals and admissions to hospital. CPRD covers 9% of the UK population and is broadly representative of the wider population [10]. The database includes diagnoses, prescriptions and tests from primary care, referrals to specialists, and diagnoses and outcomes from secondary care, which are fed back to GPs. Lifestyle and anthropometric measurements are also recorded, and linked deprivation data based on residential area are available for a subset of patients.

### Study Population

We used prescription data from CPRD to identify all male patients over the age of 18 y with incident exposure to a PDE5 inhibitor from 1 July 1999 to 1 August 2014 inclusive. This study end date was chosen because the regulations for prescribing sildenafil within the National Health Service (England) were substantially changed on 1 August 2014 [11].

PDE5 inhibitor exposure was considered incident if there were at least 12 mo of follow-up within CPRD prior to the first prescription record. Follow-up began at the date of first PDE5 inhibitor prescription (hereafter the “index date”). Exposed patients were matched to up to four unexposed male controls with at least 12 mo of follow-up in CPRD prior to the index date of the exposed patient. Exposed and control patients were matched on age (within 3 y in either direction), general practice, and diabetes status, and were required to be registered and under follow-up in CPRD at the index date. Diabetes status was included as a matching factor because in preliminary analysis it was a common comorbidity among those receiving a PDE5 inhibitor, which is likely partly because ED is a common complication of diabetes and partly because UK prescribing guidelines specifically recommend that men with diabetes should be eligible to receive a PDE5 inhibitor if needed. Furthermore, diabetes and its treatment have been postulated as having links to risks of a number of cancers including malignant melanoma [12,13]. All matching variables were measured at the index date. The index date for control patients was set to be the same as that of their exposed matches. We excluded patients with any cancer diagnoses prior to their index date and patients with no GP consultations in the year preceding their index date, since such patients may not be actively receiving care from their officially registered GP. Individuals selected as controls could later go on to start a PDE5 inhibitor; in this situation they were censored as a control at the time of starting a PDE5 inhibitor and contributed separately as an exposed patient from that time point forward (with their own matched controls).

### Exposure and Outcome

Our primary exposure was ever use of a PDE5 inhibitor, based on prescription codes (code list in S1 Text). In secondary analyses, we also investigated the effect of the time-updated cumulative number of PDE5 inhibitor prescriptions (1, 2–4, 5–9, 10–19, 20+) and time-updated years since first prescription (≤0.5, >0.5–1, >1–2, >2–4, >4 y). In a further secondary analysis, differences by specific PDE5 inhibitor drug (sildenafil, tadalafil, vardenafil) were investigated by fitting a model with a multi-category exposure variable capturing specific drug (based on the drug used in the first prescription), and comparing this to the original model (with a binary exposure variable capturing any PDE5 inhibitor use) using the likelihood ratio test.

The primary outcome was incident malignant melanoma. Clinical diagnoses are identified in UK primary care data by National Health Service Read codes in patients’ clinical records. We used Read codes mapping to ICD-10 code C43 (malignant melanoma of skin; code list in S2 Text), as identified for previous work [14]. The final code list was checked by a consultant dermatologist (S. M. L.). To further assess causality, we looked at three control outcomes not expected to be associated with PDE5 inhibitor exposure: basal cell carcinoma and solar keratosis (both associated with sun exposure) and colorectal cancer.

### Statistical Analysis

Observation time began at the index date and ended at the earliest of the following: incident melanoma, diagnosis of a cancer other than melanoma, death, transfer out of CPRD, prescription of a PDE5 inhibitor (for unexposed controls), or the end of the study period. Prior to exploring the relationship between PDE5 inhibitors and melanoma, the distributions of baseline characteristics of exposed and unexposed patients were described.

The association between the primary exposure variable and incident melanoma was then estimated using a Cox regression model with an underlying age timescale, stratified by matched set to account for the matching on age, diabetes status, and GP practice [15]. We then adjusted further for smoking status (current smoker, ex-smoker, never smoker), alcohol use (current drinker, ex-drinker, non-drinker), body mass index (BMI) (<25, 25–29, 30–34, ≥35 kg/m^2^), and number of GP consultations in the year before the index date (as a proxy for the amount of contact with health professionals and therefore opportunity for diagnosis, categorised as 1, 2–4, 5–10, ≥11 consultations). To account for any correlation due to unexposed controls appearing in the cohort more than once because they later became exposed, we used robust standard errors to adjust for clustering by the unique patient identifier variable. People with missing data for the BMI, smoking status, or alcohol use variables (13% overall) were excluded (complete case analysis), which is valid in a regression context if missingness is conditionally independent of the outcome [16]; in this context, this means we assumed that there was no association between having complete data on BMI, smoking status, and alcohol use and developing malignant melanoma, after accounting for measured covariates. Whilst this is an untestable assumption, we believe this to be more plausible in this case than the “missing at random” assumption required for multiple imputation, since recording of lifestyle-related variables may depend directly on the variable values (e.g., people with healthy BMI may be less likely to have their BMI recorded) [17]. In a secondary analysis, we further adjusted for index of multiple deprivation (IMD) (categorised in quintiles) among the subset of patients with this information available; IMD is a measure of socioeconomic status based on residential area that combines small-area deprivation data on income, employment, education, health and disability, crime, barriers to housing and services, and living environments into an overall score [18]; a higher score/quintile indicates a higher level of deprivation. To explore possible effect modification, interaction terms were fitted to generate results stratified by region within the UK (grouped by latitude into North, Midlands, and South), IMD quintile, smoking status, and current (time-updated) age group (<50, 50–59, 60–69, 70–79, ≥80 y). The main analysis was repeated for the negative control outcomes of basal cell carcinoma, colorectal cancer, and solar keratosis.

#### Sensitivity analyses

The first 12 mo of follow-up after the index date were excluded in both exposed and unexposed patients as a sensitivity analysis, to decrease the chance of reverse causality (undiagnosed melanomas leading to ED and thus PDE5 inhibitor exposure, or “protopathic bias” [19]). We also repeated the analysis restricted to patients with diabetes because previous UK prescribing guidelines stated that all patients in this group with ED were eligible for a PDE5 inhibitor prescription [20].

#### Post hoc analysis to assess residual confounding by sun exposure

Since our main analyses suggested an increased risk of all sun-exposure-related outcomes among PDE5 inhibitor users, and we had insufficient information in the dataset to directly control for confounding by sun exposure, we carried out a post hoc analysis aimed at assessing whether there may be potential for residual confounding due to sun exposure. We treated the matched exposed and unexposed groups as a nested case-control dataset in which the *outcome* was PDE5 inhibitor use. We then used conditional logistic regression to estimate the association between prior solar keratosis (a known marker of high sun exposure) and starting a PDE5 inhibitor. Unadjusted and fully adjusted odds ratios (ORs) (adjusting for all previous confounders) and 95% confidence intervals were calculated.

Observational studies using CPRD primary care data have ethical clearance subject to the approval of a research protocol by the Independent Scientific Advisory Committee for MHRA Database Research. Our approved prespecified protocol (number 15_091) is presented in S3 Text. The study was also approved by the London School of Hygiene & Tropical Medicine ethics committee (approval number 10032).

## Results

A total of 174,430 men aged ≥18 y with an incident PDE5 inhibitor prescription in the study period were identified, and 148,207 were eligible for inclusion (S1 Fig). The majority of exclusions were due to individuals having had cancer before their first PDE5 inhibitor prescription (*n =* 16,714) or having no GP consultations in the year before exposure (*n =* 9,462). Of these 148,207 exposed patients, 145,104 (98%) had eligible matches and were included, and 135,589 patients were matched to the maximum four controls. A total of 5.8% of the unexposed controls (*n =* 40,633) were censored as controls during follow-up and included in the exposed group, due to being prescribed a PDE5 inhibitor. The characteristics of the exposed and matched unexposed patients are shown in Table 1. A slightly larger proportion of exposed patients were overweight, current smokers or ex-smokers, and current alcohol drinkers, compared with unexposed controls.

**Table 1:** Characteristics of study population.

Characteristic	PDE5 Inhibitor		Total (*n =* 706,037)
	Exposed (*n =* 145,104)	Unexposed (*n =* 560,933)	
**Age (years)** *			
<40	13,899 (9.6%)	54,115 (9.6%)	68,014 (9.6%)
40–49	27,355 (18.9%)	106,105 (18.9%)	133,460 (18.9%)
50–59	44,351 (30.6%)	170,067 (30.3%)	214,418 (30.4%)
60–69	42,331 (29.2%)	162,773 (29.0%)	205,104 (29.1%)
70–79	15,502 (10.7%)	61,189 (10.9%)	76,691 (10.9%)
≥80	1,666 (1.1%)	6,684 (1.2%)	8,350 (1.2%)
Median (IQR)	57 (49–65)	57 (49–65)	57 (49–65)
**BMI (kg/m** ^**2**^ **)**			
<18	470 (0.3%)	2,997 (0.5%)	3,467 (0.5%)
18–24	35,542 (24.5%)	150,244 (26.8%)	185,786 (26.3%)
25–29	60,572 (41.7%)	218,823 (39%)	279,395 (39.6%)
30–34	28,287 (19.5%)	96,566 (17.2%)	124,853 (17.7%)
≥35	11,097 (7.6%)	39,643 (7.1%)	50,740 (7.2%)
Missing	9,136 (6.3%)	52,660 (9.4%)	61,796 (8.8%)
Median (IQR)	27 (25–31)	27 (24–30)	27 (25–30)
**Smoking status**			
Never smoker	41,283 (28.5%)	185,134 (33.0%)	226,417 (32.1%)
Current smoker	44,470 (30.6%)	164,094 (29.3%)	208,564 (29.5%)
Ex-smoker	58,777 (40.5%)	202,812 (36.2%)	261,589 (37.1%)
Missing	574 (0.4%)	8,893 (1.6%)	9467 (1.3%)
**Alcohol use**			
Non-drinker	9,221 (6.4%)	41,769 (7.4%)	50,990 (7.2%)
Current drinker	118,538 (81.7%)	436,412 (77.8%)	554,950 (78.6%)
Ex-drinker	8,537 (5.9%)	30,522 (5.4%)	39,059 (5.5%)
Missing	8,808 (6.1%)	52,230 (9.3%)	61,038 (8.6%)
**Diabetes at index date** * ^†^			
Yes	27,659 (19.1%)	91,214 (16.3%)	118,873 (16.8%)
No	117,445 (80.9%)	469,719 (83.7%)	587,164 (83.2%)
**IMD quintile**			
1 (least deprived)	22,377 (15.4%)	87,238 (15.6%)	109,615 (15.5%)
2	22,149 (15.3%)	85,061 (15.2%)	107,210 (15.2%)
3	18,802 (13.0%)	72,027 (12.8%)	90,829 (12.9%)
4	16,203 (11.2%)	62,043 (11.1%)	78,246 (11.1%)
5 (most deprived)	12,337 (8.5%)	47,065 (8.4%)	59,402 (8.4%)
Missing^‡^	53,236 (36.7%)	207,499 (37.0%)	260,735 (36.9%)
**Practice region** ^§^			
North	41,751 (28.8%)	160,691 (28.6%)	202,442 (28.7%)
Midlands	45,477 (31.3%)	175,986 (31.4%)	221,463 (31.4%)
South	57,876 (39.9%)	224,256 (40.0%)	282,132 (40.0%)
**PDE5 inhibitor**			
Sildenafil	106,091 (73.1%)	0 (0%)	106,091 (15%)
Tadalafil	31,772 (21.9%)	0 (0%)	31,772 (4.5%)
Vardenafil	7,241 (5%)	0 (0%)	7,241 (1%)
No prescriptions	0 (0%)	560,933 (100%)	560,933 (79.4%)

Data are number (percent) unless otherwise indicated.

*Matching variables.

^†^The small percent difference in the proportion exposed according to diabetes in these aggregate data, despite matching, is explained by fewer exposed diabetes patients being matched to the maximum four controls, compared to those without diabetes.

^‡^Linked individual-level deprivation data available only for English general practices participating in the data linkage scheme.

^§^North = North East England, North West England, Yorkshire, and Scotland; Midlands = East Midlands, West Midlands, East of England, Wales; South = South West England, South Central England, London, South East England. Note that general practice was a matching factor, so the distribution of practice region is similar between the exposed and unexposed by design.

During 3.44 million person-years of follow-up (mean 4.9 y/person), the crude incidence rate of melanoma was higher in PDE5 inhibitor users than in unexposed individuals (43.7 versus 36.8 per 100,000 person-years; Table 2). The unadjusted hazard ratio (HR), accounting for matched variables and restricted to patients with no missing data on BMI, smoking status, and alcohol use, showed weak evidence of an increased risk of melanoma in exposed patients in comparison to unexposed patients (HR = 1.16, 95% CI 1.03–1.31). There was little change in the estimate when adjusting for all potential confounders (adjusted HR = 1.14, 95% CI 1.01–1.29, *p =* 0.04). When further adjusted for IMD quintile in the subset of 445,302 patients (63%) with this information available, the observed association was smaller but the confidence interval was wider, as expected (HR = 1.11, 95% CI 0.96–1.29, *p =* 0.17).

**Table 2:** Crude rate for malignant melanoma and control outcomes by exposure to PDE5 inhibitors, and unadjusted and adjusted hazard ratios.

Outcome by Exposure	Number of Events	Person-Years of Follow-Up (100,000s)	Crude Rate (per 100,000 Person-Years)	Unadjusted HR* (95% CI)	Adjusted HR^†^ (95% CI)	*p*-Value
***Primary outcome***						
**Malignant melanoma**						0.04
Ever exposed	321	7.4	43.7 (39.1, 48.7)	1.16 (1.03, 1.31)	1.14 (1.01, 1.29)	
Unexposed	994	27.0	36.8 (34.5, 39.1)			
***Control outcomes***						
**Basal cell carcinoma**						<0.001
Ever exposed	3,257	7.4	443.0 (428.0, 458.5)	1.18 (1.14, 1.23)	1.15 (1.11, 1.19)	
Unexposed	9,801	27.0	362.6 (355.5, 369.9)			
**Solar keratosis**						<0.001
Ever exposed	4,408	7.0	626.2 (608.0, 644.9)	1.25 (1.21, 1.29)	1.21 (1.17, 1.25)	
Unexposed	12,831	26.2	490.6 (482.2, 499.1)			
**Colorectal cancer**						0.01
Ever exposed	879	7.4	119.6 (111.9, 127.7)	0.92 (0.86, 0.99)	0.91 (0.85, 0.98)	
Unexposed	3,304	27.0	122.2 (118.1, 126.5)			

*Cox model with age timescale, stratified by matched set, excluding those with missing data on BMI, smoking status, or alcohol use (*n =* 88,599/706,037; 12.5%).

^†^Cox model with age timescale, stratified by matched set, excluding those with missing data on BMI, smoking status, or alcohol use (*n =* 88599/706019, 12.5%), and adjusted for the following (with HR [95% CI] in final model): number of consultations in year before index date (1 [reference], 2–4 [1.07 (0.87–1.32)], 5–10 [1.12 (0.91–1.38)], ≥11 [1.30 (1.03–1.63)]), BMI category (<25 kg/m^2^ [reference], 25–29 kg/m^2^ [1.10 (0.95–1.26)], 30–34 kg/m^2^ [1.12 (0.93–1.35)], ≥35 kg/m^2^ [1.10 (0.83–1.45)]), alcohol use (non-drinker [reference], current drinker [1.31 (1.02–1.67)], ex-drinker [1.06 (0.74–1.51)]), smoking status (never smoker [reference], current smoker [0.73 (0.62–0.86)], ex-smoker [0.91 (0.79–1.05)]).

### Analysis of Negative Control Outcomes

For the two negative control outcomes related to sun exposure, basal cell carcinoma and solar keratosis, we estimated similar HRs to those observed in the main malignant melanoma analysis (adjusted HR = 1.15, 95% CI 1.11–1.19, *p <* 0.001, and HR = 1.21, 95% CI 1.17–1.25, *p <* 0.001, respectively; Table 2), but there was no evidence of any increased risk of colorectal cancer among PDE5 inhibitor users (HR = 0.91, 95% CI 0.85–0.98, *p =* 0.01).

### Effect of Cumulative Exposure and Effect Modification by Individual-Level Factors

**Fig 1:**
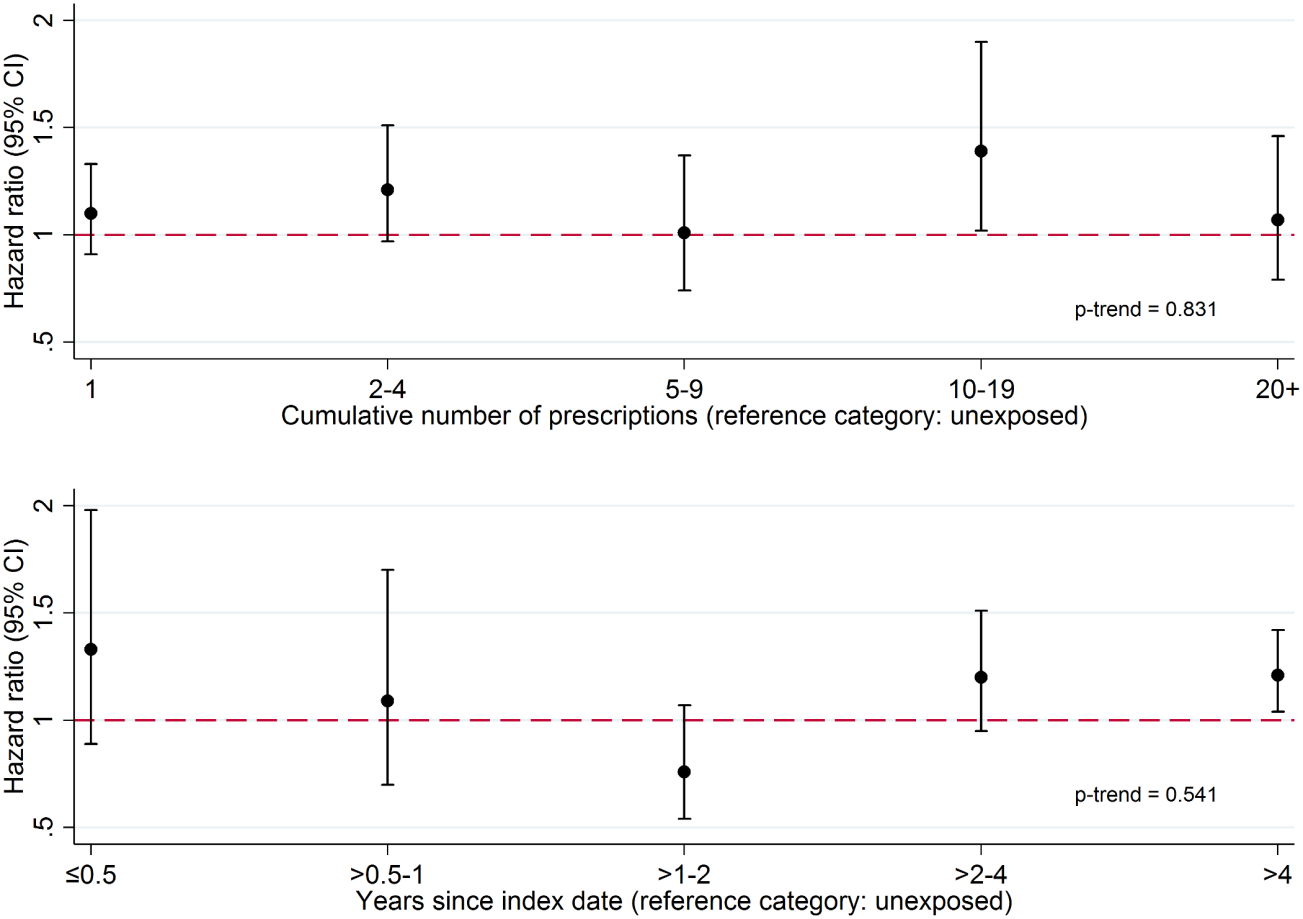
Association between PDE5 inhibitor use and malignant melanoma, by cumulative number of prescriptions received and time since first prescription (index date). Cumulative number of prescriptions received (top panel) and time since index date (bottom panel). From a Cox model with age timescale, stratified by matched set and adjusted for number of consultations in year before index date, BMI category, alcohol use, and smoking status.

**Fig 2:**
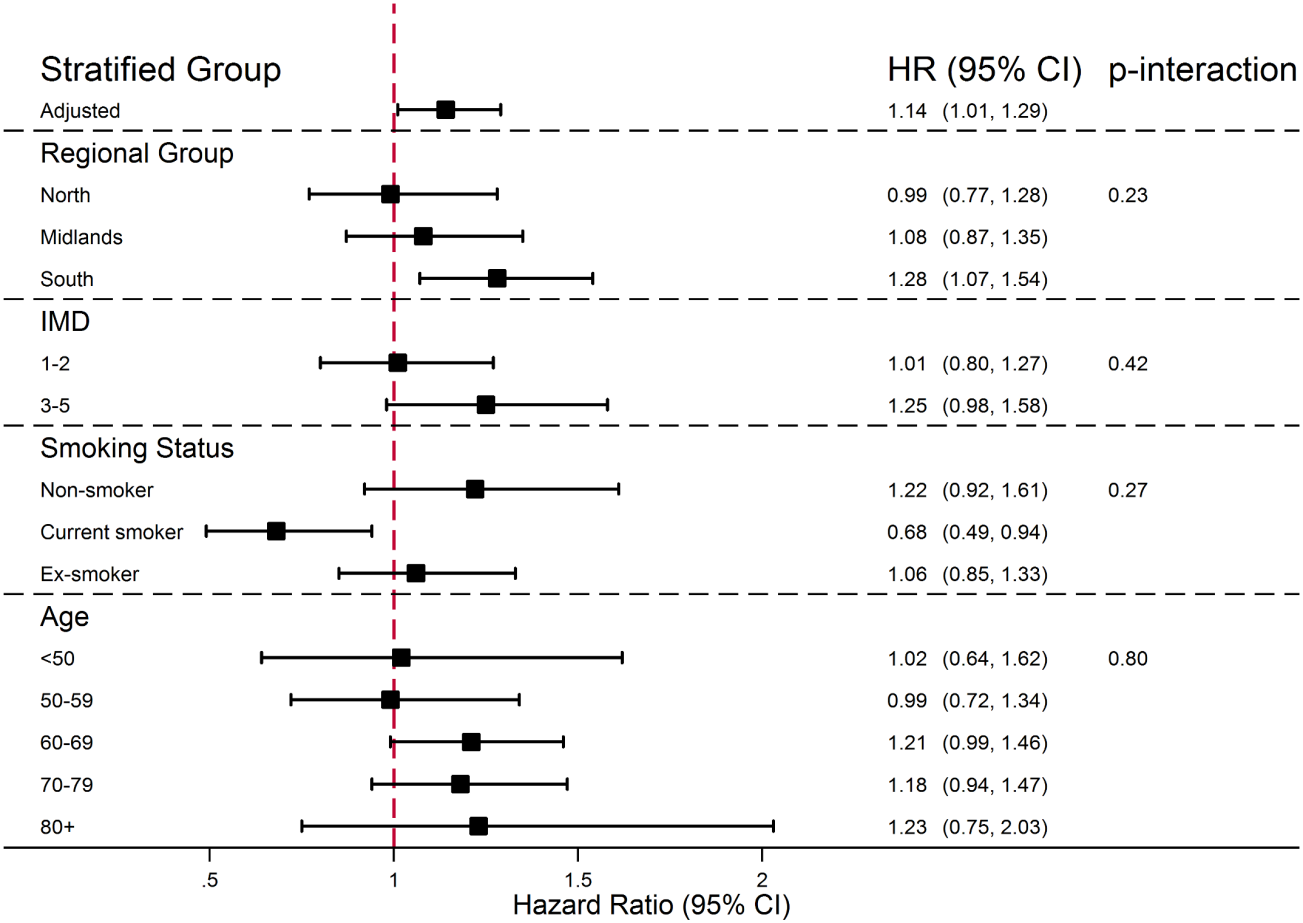
Effect of PDE5 inhibitor use on malignant melanoma risk, stratified by individual-level factors. From Cox models containing interaction terms between individual-level factors and exposure (interactions fitted one at a time), with age timescale, stratified by matched set and adjusted for number of consultations in year before index date, BMI category, alcohol use, and smoking status.

There was no evidence that the association between PDE5 inhibitor use and melanoma risk increased with either cumulative number of PDE5 inhibitor prescriptions received or number of years since first prescription (Fig 1; *p*-trend = 0.83 and *p =* 0.54, respectively). Stratified analyses are presented in Fig 2; point estimates for the association between PDE5 inhibitor use and melanoma risk were somewhat higher for individuals in the South region, for those with higher deprivation, for non-smokers, and among those aged ≥80 y, but these interactions were compatible with chance variation (*p* ≥ 0.23 in all cases).

### Effect by Specific PDE5 Inhibitor Drug

There was no evidence that the association between PDE5 inhibitor use and melanoma risk differed by specific PDE5 inhibitor drug (*p =* 0.42), but the number of melanoma outcomes among men on drugs other than sildenafil was small (*n =* 50 and *n =* 12 events for tadalafil and vardenafil, respectively), leading to limited power.

### Sensitivity Analyses

In sensitivity analyses, the observed increase in risk of melanoma among PDE5 inhibitor users was of similar magnitude to that estimated in the main analysis when the analysis was restricted to those diagnosed with diabetes prior to index date (HR = 1.11, 95% CI 0.81–1.52, *p =* 0.51) and when the first year after the index date was excluded from analysis (HR = 1.13, 95% CI 0.99–1.29, *p =* 0.07), though—as expected due to the reduced numbers—confidence intervals were wider.

### Post Hoc Analysis—Association between Prior Solar Keratosis and Initiating a PDE5 Inhibitor

Adjusted conditional logistic regression showed overwhelming evidence of a positive association between having a prior diagnosis of solar keratosis and initiating a PDE5 inhibitor (OR = 1.28, 95% CI 1.23–1.34, *p <* 0.001; Table 3).

**Table 3:** Post hoc analysis of association between prior solar keratosis and initiation of a PDE5 inhibitor.

Exposure	Initiated PDE5 Inhibitor	Did Not Initiate PD5 Inhibitor	Unadjusted OR* (95% CI)	Adjusted OR^†^ (95% CI)	*p*-Value
Prior solar keratosis	3,630	11,093	1.30 (1.25, 1.35)	1.28 (1.23, 1.34)	<0.001
No prior solar keratosis	141,474	549,840			

*From conditional logistic regression of matched sets with outcome of initiating PDE5 inhibitor.

^†^From conditional logistic regression of matched sets with outcome of initiating PDE5 inhibitor, adjusted for BMI, alcohol use, and smoking status.

## Discussion

In this large population-based matched cohort study, we found weak evidence of a positive association between exposure to a PDE5 inhibitor and risk of malignant melanoma after matching or adjusting for key potential confounders. However, further analyses strongly suggested that this observed association was non-causal and explained by greater sun exposure among PDE5 inhibitor users. A number of observations support this conclusion. First, among those exposed to a PDE5 inhibitor, there was no increase in risk with a greater cumulative number of PDE5 inhibitor prescriptions received or with longer time since first exposure. Second, the association was non-specific: in negative control analyses, a similar increased risk of two other sun-exposure-related outcomes (basal cell carcinoma and solar keratosis) was observed among PDE5 inhibitor users. There was no increase in risk for colorectal cancer, which is unrelated to sun exposure. Third, we found strong evidence in a post hoc analysis that PDE5 inhibitor users were more likely to have had solar keratosis prior to their first PDE5 inhibitor prescription, suggesting that PDE5 inhibitor users were more likely to have experienced excess sun or UV exposure than non-users, even before starting a PDE5 inhibitor.

### Strengths and Limitations of the Study

This large study included all patients with an incident PDE5 inhibitor prescription in CPRD over a 15-y period, so we had high power to detect even a relatively small effect size. We used a number of approaches to assess causality, including investigating whether there was a dose-response relationship with regard to the number of prescriptions received, using several negative control outcomes, and investigating evidence for pre-exposure differences in sun/UV exposure. By using primary care data, we were able to include data on important potential confounders related to lifestyle and socioeconomic status, namely, smoking status, alcohol use, deprivation, and BMI. We conducted a number of sensitivity analyses to assess the robustness of our findings.

There were also important limitations to our study. First, we had no patient-level data on sun/UV exposure, which is the most important known non-genetic factor associated with malignant melanoma. Patients were matched on general practice and therefore on geographical area of residence; this is likely to have reduced any differences in sun exposure related to region, but there may still have been differences in amount of time spent outdoors, number of foreign holidays, and use of tanning beds. Our secondary analysis, which adjusted for a measure of deprivation based on residential area for patients with available data, is likely to have reduced residual confounding since people from the same socioeconomic group are more likely to be similar in terms of these characteristics. Given the lack of any direct data on sun and UV exposure, we made use of data on other outcomes known to be related to sun exposure to assess the likely impact of residual confounding: we investigated basal cell carcinoma and solar keratosis as negative control outcomes, and we looked at differences in prior solar keratosis between exposed and unexposed patients to investigate whether sun exposure might be associated with prescription of a PDE5 inhibitor. We also lacked information regarding skin type and family history of melanoma, which are both known to be associated with melanoma. Furthermore, due to the low number of patients prescribed tadalafil and vardenafil, we had limited power to detect differences in the associations between specific PDE5 inhibitor drugs and melanoma risk.

Another important limitation is the potential for misclassified exposure status. Primary care prescriptions are well captured in CPRD because, in nearly all cases, prescriptions are issued electronically and therefore recorded automatically. However, there are no data on whether a patient has actually filled the assigned prescription or taken the drug. Furthermore, PDE5 inhibitors may have been purchased without prescription by some patients included as controls. It is also possible that men in the control group could have received a PDE5 inhibitor prescription from a specialist outside primary care. To our knowledge, there are no published data on the extent of online purchasing of PDE5 inhibitors or on the prescribing of these drugs by specialists, but under the UK healthcare system we believe that the vast majority of routine PDE5 inhibitor prescriptions would have been issued within primary care. Nevertheless, these forms of exposure misclassification could have led to an underestimation of any real difference in outcomes between exposed and unexposed patients. Although we were unable to distinguish between therapeutic indications for these medications, we included only PDE5 inhibitor formulations that are recommended for the treatment of ED in the British National Formulary [1] in order to obtain a relatively homogeneous study population in terms of drug indication. Due to overlapping indications, we cannot be sure that all of the PDE5 inhibitors were prescribed for treatment of ED; however, it is worth noting that any causal effect of PDE5 inhibitors on melanoma risk would be expected to be independent of the clinical indication, so one would not expect the inclusion of a minority of men with other indications to lead to serious underestimation of any true causal effect.

Patients in CPRD are broadly representative of the wider UK population. However, during the study period, PDE5 inhibitors were subsidised by the National Health Service only for people specifically experiencing distress due to ED or whose ED was linked to specific comorbidities, and this could limit the ability to generalise our findings to all PDE5 inhibitor users. However, our preliminary descriptive analyses suggested that these drugs were prescribed widely to individuals who did not have any record of specific qualifying medical conditions, likely justified by patient “distress”, which qualified men to receive the drug and could have been interpreted widely. So, in practice, it is likely that patients across the clinical spectrum were represented in the study. Furthermore, if there were a true causal increase in risk of melanoma with PDE5 inhibitor use, it is likely that this would be observed even in a clinically selected population. We would caution against generalising our results to populations with a substantially different ethnic mix to the UK population, since ethnicity is likely to be an important predictor of melanoma risk. We had insufficient data on ethnicity to investigate this as an effect modifier.

Melanoma outcomes could have been misclassified if cancer diagnoses in CPRD were not reliable. However, a recent validation study found that 87% of melanoma cases reported in CPRD were also recorded in cancer registries and that 96% of melanoma cases reported in cancer registries were also reported in CPRD [21], suggesting minimal misclassification. We did not have data on melanoma stage at diagnosis so were unable to assess associations between PDE5 inhibitor exposure and melanoma of different stages.

### Comparison with Other Studies

The present study is, to our knowledge, the largest to date to investigate the association between PDE5 inhibitor use and malignant melanoma risk, and the strength of association that we observed was consistent with that found by Loeb et al. in a recent study using Swedish registry data (OR for PDE5 inhibitor use: 1.21, 95% CI 1.08–1.36) [6] and considerably smaller than the association reported by Li et al. in the 2014 US cohort study that originally raised concerns on this topic (HR for sildenafil use: 1.84, 95% CI 1.04–3.22) [5]. Our findings, along with those from the aforementioned Swedish registry study, would appear to rule out a true association of the magnitude originally reported by Li et al., since even at the upper 95% confidence limits, estimates from the two more recent studies are incompatible with anything greater than a 29%–36% increase in risk. As with the Swedish registry study, we found an association between PDE5 inhibitor exposure and basal cell carcinoma. We also included colorectal cancer as a negative control outcome unrelated to sun exposure and found no association with PDE5 inhibitor use, suggesting that differential ascertainment due to health seeking behaviours or healthcare contact is unlikely to explain the increase in observed risk of melanoma. Like Loeb et al., we found no relationship between the number of PDE5 inhibitor prescriptions and melanoma risk, which argues against a causal relationship. Our post hoc finding of an association between prior solar keratosis diagnosis and initiation of a PDE5 inhibitor adds a unique insight, which we think makes a compelling case for the observed association being driven by confounding by sun or UV exposure. Our study population and data source have similarities with those used by Loeb et al.; in both cases, a large European population-based cohort was derived from electronic health records. One notable difference was that we observed a less striking socioeconomic gradient among PDE5 inhibitor users than Loeb et al. observed. However, unlike in the Swedish data source, which included individual-level information on socioeconomic status, the deprivation measure that we used was based on patient postal district only. Perhaps more importantly, PDE5 inhibitors are subsidised by the National Health Service in the UK, unlike in Sweden, so one would not expect as strong a relationship between socioeconomic factors and PDE5 inhibitor use. In contrast to the two European studies, the original study by Li et al. used a much smaller dataset from a specific cohort of US health professionals. Exposure to sildenafil was self-reported as recent use at a single point in calendar time, and only those answering the question were included, introducing a potential for both misclassification of exposure and selection bias. Outcomes were also self-reported from biennial surveys. However, a unique strength of that study was inclusion of data relating to UV exposure, which should have reduced confounding, though these data were also self-reported and likely to have been prone to reporting error. One point worth noting is that Li et al. did not find an increased risk of basal cell carcinoma, which would be expected if confounding by sun exposure explained their findings for melanoma. If, as our results strongly suggest, there is no true causal association between PDE5 inhibitor use and melanoma, then the findings from the study by Li et al. may have been driven by some other bias mechanism.

### Conclusion

This large matched cohort study using data from UK primary care strongly suggests that the previously reported association between PDE5 inhibitors and malignant melanoma is not causal. Consistent with recent data from Sweden [6], we found weak evidence of a small increased risk of melanoma among PDE5 inhibitor users in our primary analysis; however, greater exposure did not appear to be associated with higher risk, the association was not specific to melanoma and was also observed for other sun-exposure-related conditions, and there was strong evidence that exposed patients were more likely to have had high sun or UV exposure, even before their first PDE5 inhibitor prescription.

## Supporting Information

S1 Fig Participant flow diagram. (TIF)

S1 STROBE Statement Checklist of items that should be included in reports of observational studies. (DOC)

S1 Text List of CPRD product codes used to identify PDE5 inhibitor prescriptions. (DOCX)

S2 Text List of National Health Service Read codes used to identify malignant melanoma. (DOCX)

S3 Text Prespecified and approved study protocol and explanation for changes. (DOCX)

## Abbreviations

BMI: body mass index
CPRD: Clinical Practice Research Datalink
ED: erectile dysfunction
GP: general practitioners
HR: hazard ratio
IMD: index of multiple deprivation
OR: odds ratio
PDE5: phosphodiesterase type 5
