# Supplementary figures and images for: Phosphodiesterase Type 5 Inhibitors and Risk of Malignant Melanoma: Matched Cohort Study Using Primary Care Data from the UK Clinical Practice Research Datalink
Source: PLoS Med. 2016 Jun 14;13(6):e1002037. doi: 10.1371/journal.pmed.1002037 (PMC4907438; doi:10.1371/journal.pmed.1002037)

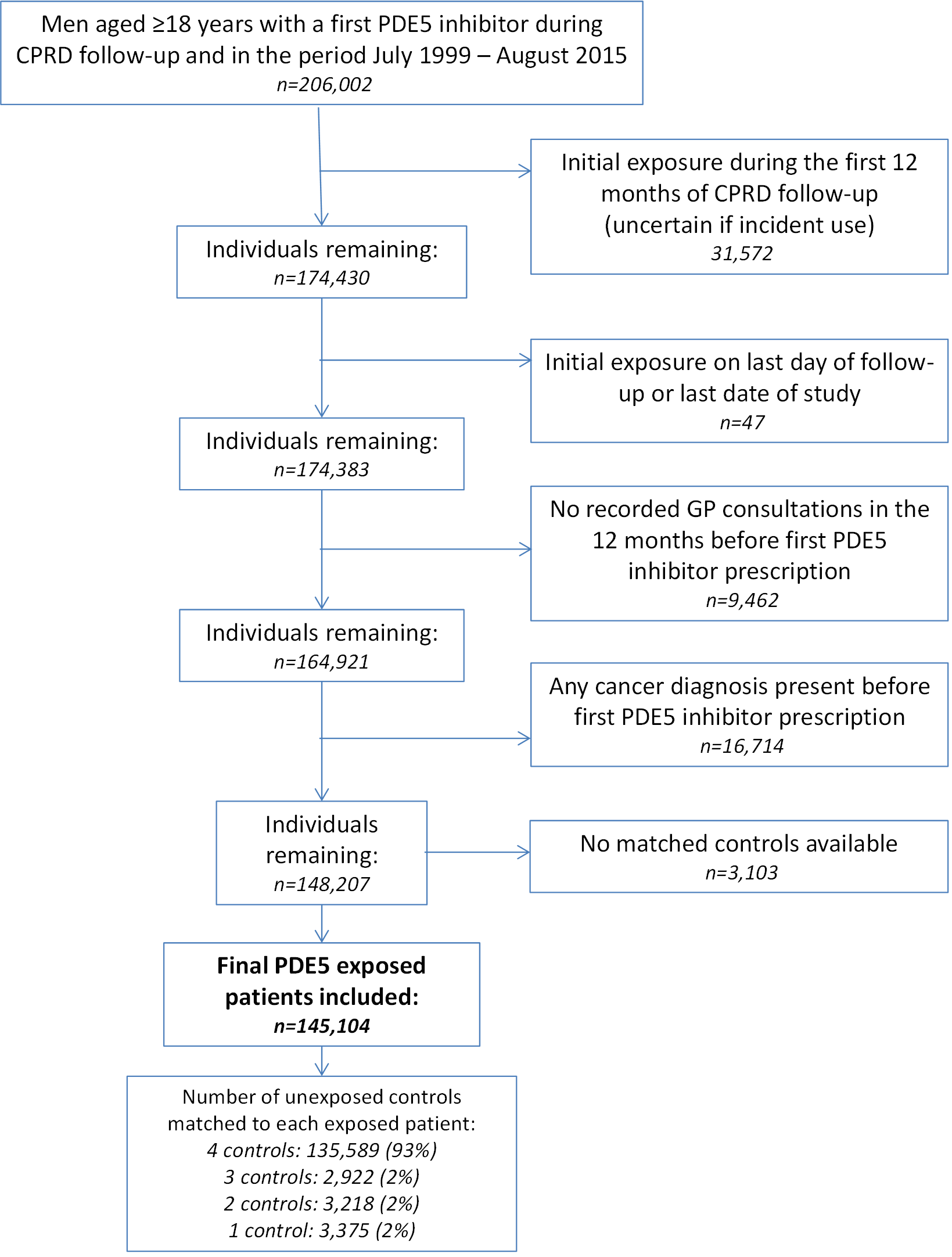

Supplement: S1 Fig — (TIF) [file pmed.1002037.s001.tif]
