# Supplementary material for: Phosphodiesterase Type 5 Inhibitors and Risk of Malignant Melanoma: Matched Cohort Study Using Primary Care Data from the UK Clinical Practice Research Datalink
Source: PLoS Med. 2016 Jun 14;13(6):e1002037. doi: 10.1371/journal.pmed.1002037 (PMC4907438; doi:10.1371/journal.pmed.1002037)
Supplement: S1 STROBE Statement — (DOC) [file pmed.1002037.s002.doc]

STROBE Statement—checklist of items that should be included in reports of observational studies

|  | Item No | Recommendation |
| --- | --- | --- |
| **Title and abstract** | 1 | (*a*) Indicate the study’s design with a commonly used term in the title or the abstract  **Title** |
|  |  | (*b*) Provide in the abstract an informative and balanced summary of what was done and what was found  **Abstract** |
| Introduction | | |
| Background/rationale | 2 | Explain the scientific background and rationale for the investigation being reported  **Introduction paragraphs 1-2 (previous lab and epidemiological evidence outlined)** |
| Objectives | 3 | State specific objectives, including any prespecified hypotheses  **Introduction paragraph 3 “we aimed to examine the association between PDE5 inhibitors used for erectile dysfunction and the risk of incident melanoma in a large cohort of men using data from UK primary care, and to assess causality of any observed increase in risk.”** |
| Methods | | |
| Study design | 4 | Present key elements of study design early in the paper  **Methods, Study design and data source, paragraph 1: “We carried out a matched cohort study using prospectively collected data from the UK Clinical Practice Research Datalink (CPRD)”; Methods, Study population; Methods, Exposure and outcome** |
| Setting | 5 | Describe the setting, locations, and relevant dates, including periods of recruitment, exposure, follow-up, and data collection  **Methods, Study design and data source, paragraph 1: “using prospectively collected data from the UK Clinical Practice Research Datalink (CPRD), a database containing anonymised primary care data from general practitioners”; Methods, Study population, paragraph 1: “all male patients over the age of 18 with incident exposure to a PDE5 inhibitor from 1st July 1999 to 1st August 2014 inclusive”** |
| Participants | 6 | (*a*) *Cohort study*—Give the eligibility criteria, and the sources and methods of selection of participants. Describe methods of follow-up  **Methods, Study design and data source, paragraph 1: “using prospectively collected data from the UK Clinical Practice Research Datalink (CPRD)…. The database includes diagnoses, prescriptions and tests from primary care, referrals to specialists, as well as diagnoses and outcomes from secondary care, which are fed back to general practitioners.”; Methods, Study population, paragraph 1: “all male patients over the age of 18 with incident exposure to a PDE5 inhibitor from 1st July 1999 to 1st August 2014 inclusive”**  *Case-control study*—Give the eligibility criteria, and the sources and methods of case ascertainment and control selection. Give the rationale for the choice of cases and controls  *Cross-sectional study*—Give the eligibility criteria, and the sources and methods of selection of participants |
|  |  | (*b*) *Cohort study*—For matched studies, give matching criteria and number of exposed and unexposed  **Methods, Study population, paragraph 2: “Exposed patients were matched to up to four male controls with at least 12 months follow-up in CPRD prior to the index date of the exposed patient. Exposed and control patients were matched on age (within 3 years in either direction), GP practice, diabetes status, and active registration at the index date.”**  *Case-control study*—For matched studies, give matching criteria and the number of controls per case |
| Variables | 7 | Clearly define all outcomes, exposures, predictors, potential confounders, and effect modifiers. Give diagnostic criteria, if applicable  **Methods, Exposure and outcome; Methods, Statistical analysis, paragraph 2: “We then adjusted further for smoking status (current smoker, ex-smoker, never smoker), alcohol use (current drinker, ex drinker, non-drinker) body mass index (BMI, <25, 25-29, 30-34, ≥35 kg/m2), and number of consultations in the year before the index date (as a proxy for amount of contact with health professionals and therefore opportunity for diagnosis, categorised as 1, 2-4, 5-10, ≥11 consultations).”** |
| Data sources/ measurement | 8* | For each variable of interest, give sources of data and details of methods of assessment (measurement). Describe comparability of assessment methods if there is more than one group  **Methods, Study design and data source, paragraph 1: “using prospectively collected data from the UK Clinical Practice Research Datalink (CPRD)… The database includes diagnoses, prescriptions and tests from primary care, referrals to specialists, as well as diagnoses and outcomes from secondary care, which are fed back to general practitioners. Lifestyle and anthropometric measurements are also recorded and linked deprivation data based on residential area are available for a subset of patients.”** |
| Bias | 9 | Describe any efforts to address potential sources of bias  **Methods, Statistical analysis, paragraph 2; Methods, Statistical analysis, Sensitivity analyses; Methods, Statistical analysis, Post hoc analysis to assess residual confounding by sun exposure section.** |
| Study size | 10 | Explain how the study size was arrived at  **N/a all available data used (as stated in Methods, Study population, paragraph 1)** |
| Quantitative variables | 11 | Explain how quantitative variables were handled in the analyses. If applicable, describe which groupings were chosen and why  **Methods, Exposure and outcome; Methods, Statistical analysis, paragraph 2: “We then adjusted further for smoking status (current smoker, ex-smoker, never smoker), alcohol use (current drinker, ex drinker, non-drinker) body mass index (BMI, <25, 25-29, 30-34, ≥35 kg/m2), and number of consultations in the year before the index date (as a proxy for amount of contact with health professionals and therefore opportunity for diagnosis, categorised as 1, 2-4, 5-10, ≥11 consultations).”** |
| Statistical methods | 12 | (*a*) Describe all statistical methods, including those used to control for confounding  **Methods, Statistical analysis** |
|  |  | (*b*) Describe any methods used to examine subgroups and interactions  **Methods, Statistical analysis, paragraph 2: “To explore possible effect modification, interaction terms were fitted to generate results stratified by region within the UK (grouped by latitude into north, midlands and south), IMD quintile, smoking status, and current (time-updated) age group (grouped into <50, 50-59, 60-69, 70-79, >80 years).”** |
|  |  | (*c*) Explain how missing data were addressed  **Methods, Statistical analysis, paragraph 2: “People with missing data on these variables (13% overall) were excluded (complete case analysis) which is valid in a regression context if missingness is conditionally independent of the outcome;[16] in this context, this means we assumed that there was no association between having complete data on BMI, smoking and alcohol, and developing malignant melanoma, after accounting for measured covariates. Whilst this is an untestable assumption, we believed this to be more plausible in this case than the “missing at random” assumption required for multiple imputation, since recording of lifestyle-related variables may depend directly on the variable values (e.g. people with healthy BMI may be less likely to have their BMI recorded).17”** |
|  |  | (*d*) *Cohort study*—If applicable, explain how loss to follow-up was addressed  **N/a**  *Case-control study*—If applicable, explain how matching of cases and controls was addressed  *Cross-sectional study*—If applicable, describe analytical methods taking account of sampling strategy |
|  |  | (*e*) Describe any sensitivity analyses  **Methods, Sensitivity analyses** |

Continued on next page

| Results | | |
| --- | --- | --- |
| Participants | 13* | (a) Report numbers of individuals at each stage of study—eg numbers potentially eligible, examined for eligibility, confirmed eligible, included in the study, completing follow-up, and analysed  **Results, paragraph 1: “A total of 174,430 men aged ≥18 years with an incident PDE5 inhibitor prescription in the study period were identified, and 148,207 were eligible for inclusion”; and S1 Fig** |
|  |  | (b) Give reasons for non-participation at each stage  **Results, paragraph 1: “The majority of exclusions were due to having had cancer before first prescription (n=16,714), or having no GP consultations in the year before exposure (n=9,462).”; and S1 Fig** |
|  |  | (c) Consider use of a flow diagram  **S1 Fig** |
| Descriptive data | 14* | (a) Give characteristics of study participants (eg demographic, clinical, social) and information on exposures and potential confounders  **Table 1** |
|  |  | (b) Indicate number of participants with missing data for each variable of interest  **Table 1** |
|  |  | (c) *Cohort study*—Summarise follow-up time (eg, average and total amount)  **Results, paragraph 2: “During 3.44 million person-years of follow-up (mean 4.9 years/person)…”** |
| Outcome data | 15* | *Cohort study*—Report numbers of outcome events or summary measures over time  **Table 2** |
|  |  | *Case-control study—*Report numbers in each exposure category, or summary measures of exposure |
|  |  | *Cross-sectional study—*Report numbers of outcome events or summary measures |
| Main results | 16 | (*a*) Give unadjusted estimates and, if applicable, confounder-adjusted estimates and their precision (eg, 95% confidence interval). Make clear which confounders were adjusted for and why they were included  **Results, paragraph 2; Table 2 including footnotes** |
|  |  | (*b*) Report category boundaries when continuous variables were categorized  **Table 1** |
|  |  | (*c*) If relevant, consider translating estimates of relative risk into absolute risk for a meaningful time period  **N/a as analyses not considered consistent with a real causal effect** |
| Other analyses | 17 | Report other analyses done—eg analyses of subgroups and interactions, and sensitivity analyses  **Results, Analysis of negative control outcomes; Results, Effect of cumulative exposure and effect modification by individual-level factors; Results, Effect by specific PDE5 inhibitor drug; Results, Sensitivity analyses; Results, Post hoc analysis – association between prior solar keratosis and initiating a PDE5 inhibitor; Table 3** |
| Discussion | | |
| Key results | 18 | Summarise key results with reference to study objectives  **Discussion, paragraph 1: “In this large population-based matched cohort study, we found weak evidence of a positive association between exposure to a PDE5 inhibitor and risk of malignant melanoma after matching or adjusting for key potential confounders. However, further analyses strongly suggested that this observed association was non-causal, and explained by greater sun exposure among PDE5 users.”** |
| Limitations | 19 | Discuss limitations of the study, taking into account sources of potential bias or imprecision. Discuss both direction and magnitude of any potential bias  **Discussion, Strengths and limitations of study** |
| Interpretation | 20 | Give a cautious overall interpretation of results considering objectives, limitations, multiplicity of analyses, results from similar studies, and other relevant evidence  **Discussion, Conclusions** |
| Generalisability | 21 | Discuss the generalisability (external validity) of the study results  **Discussion, Strengths and limitations of study, paragraph 4: “Patients in CPRD are broadly representative of the wider UK population. However, during the study period, PDE5 inhibitors were only subsidised by the National Health Service for people specifically experiencing distress or whose erectile dysfunction was linked to specific comorbidities, and this could limit the ability to generalise our findings to all PDE5 inhibitor users. However, our preliminary descriptive analyses suggested that these drugs were prescribed widely to individuals who did not have any record of specific qualifying medical conditions…So in practice, it is likely that patients across the clinical spectrum were represented in the study…We would caution against generalising our results to populations with a substantially different ethnic mix to the UK population, since ethnicity is likely to be an important predictor of melanoma risk.”** |
| Other information | | |
| Funding | 22 | Give the source of funding and the role of the funders for the present study and, if applicable, for the original study on which the present article is based |

**Included in submission**

*Give information separately for cases and controls in case-control studies and, if applicable, for exposed and unexposed groups in cohort and cross-sectional studies.

**Note:** An Explanation and Elaboration article discusses each checklist item and gives methodological background and published examples of transparent reporting. The STROBE checklist is best used in conjunction with this article (freely available on the Web sites of PLoS Medicine at http://www.plosmedicine.org/, Annals of Internal Medicine at http://www.annals.org/, and Epidemiology at http://www.epidem.com/). Information on the STROBE Initiative is available at www.strobe-statement.org.
