# Supplementary material for: Phosphodiesterase Type 5 Inhibitors and Risk of Malignant Melanoma: Matched Cohort Study Using Primary Care Data from the UK Clinical Practice Research Datalink
Source: PLoS Med. 2016 Jun 14;13(6):e1002037. doi: 10.1371/journal.pmed.1002037 (PMC4907438; doi:10.1371/journal.pmed.1002037)
Supplement: S1 Text — (DOCX) [file pmed.1002037.s003.docx]

**S1 Text: List of CPRD product codes used to identify PDE5 inhibitor prescriptions**

6207 tadalafil 20mg tablets

61184 sildenafil 100mg tablets (teva uk ltd)

6809 vardenafil 20mg tablets

57369 sildenafil 100mg chewable tablets sugar free

1257 sildenafil 100mg tablets

6777 levitra 20mg tablets (bayer plc)

57491 sildenafil 25mg chewable tablets sugar free

704 sildenafil 25mg tablets

60333 nipatra 50mg chewable tablets (amco)

14860 levitra 5mg tablets (bayer plc)

554 sildenafil 50mg tablets

6457 levitra 10mg tablets (bayer plc)

45776 vardenafil 10mg orodispersible tablets sugar free

6203 vardenafil 5mg tablets

57411 sildenafil 50mg chewable tablets sugar free

45844 levitra 10mg orodispersible tablets (bayer plc)

1732 viagra 100mg tablets (pfizer ltd)

6214 vardenafil 10mg tablets

1452 viagra 25mg tablets (pfizer ltd)

1456 viagra 50mg tablets (pfizer ltd)

52369 levitra 10mg tablets (sigma pharmaceuticals plc)

6015 cialis 10mg tablets (eli lilly and company ltd)

48764 cialis 20mg tablets (necessity supplies ltd)

39289 tadalafil 2.5mg tablets

39285 cialis 5mg tablets (eli lilly and company ltd)

6148 cialis 20mg tablets (eli lilly and company ltd)

50233 cialis 10mg tablets (sigma pharmaceuticals plc)

49347 cialis 20mg tablets (mawdsley-brooks & company ltd)

39096 tadalafil 5mg tablets

794 tadalafil 10mg tablets

39243 cialis 2.5mg tablets (eli lilly and company ltd)
