# Supplementary material for: Phosphodiesterase Type 5 Inhibitors and Risk of Malignant Melanoma: Matched Cohort Study Using Primary Care Data from the UK Clinical Practice Research Datalink
Source: PLoS Med. 2016 Jun 14;13(6):e1002037. doi: 10.1371/journal.pmed.1002037 (PMC4907438; doi:10.1371/journal.pmed.1002037)
Supplement: S2 Text — (DOCX) [file pmed.1002037.s004.docx]

**S2 Text: List of NHS Read codes used to identify malignant melanoma**

4M71.00 CLARK MELANOMA LEVEL 2

4M72.00 CLARK MELANOMA LEVEL 3

4M73.00 CLARK MELANOMA LEVEL 4

4M74.00 CLARK MELANOMA LEVEL 5

B327500 MALIGNANT MELANOMA OF ANKLE

B322000 MALIGNANT MELANOMA OF AURICLE (EAR)

B325000 MALIGNANT MELANOMA OF AXILLA

B325700 MALIGNANT MELANOMA OF BACK

B325100 MALIGNANT MELANOMA OF BREAST

B325200 MALIGNANT MELANOMA OF BUTTOCK

B325800 MALIGNANT MELANOMA OF CHEST WALL

B323100 MALIGNANT MELANOMA OF CHIN

B322.00 MALIGNANT MELANOMA OF EAR AND EXTERNAL AURICULAR CANAL

B322z00 MALIGNANT MELANOMA OF EAR AND EXTERNAL AURICULAR CANAL NOS

B322100 MALIGNANT MELANOMA OF EXTERNAL AUDITORY MEATUS

B323000 MALIGNANT MELANOMA OF EXTERNAL SURFACE OF CHEEK

B323400 MALIGNANT MELANOMA OF EXTERNAL SURFACE OF NOSE

B323200 MALIGNANT MELANOMA OF EYEBROW

B321.00 MALIGNANT MELANOMA OF EYELID INCLUDING CANTHUS

B323z00 MALIGNANT MELANOMA OF FACE NOS

B326400 MALIGNANT MELANOMA OF FINGER

B327700 MALIGNANT MELANOMA OF FOOT

B326200 MALIGNANT MELANOMA OF FORE-ARM

B323300 MALIGNANT MELANOMA OF FOREHEAD

B327900 MALIGNANT MELANOMA OF GREAT TOE

B325300 MALIGNANT MELANOMA OF GROIN

B326300 MALIGNANT MELANOMA OF HAND

B327600 MALIGNANT MELANOMA OF HEEL

B327000 MALIGNANT MELANOMA OF HIP

B327200 MALIGNANT MELANOMA OF KNEE

B320.00 MALIGNANT MELANOMA OF LIP

B327400 MALIGNANT MELANOMA OF LOWER LEG

B327.00 MALIGNANT MELANOMA OF LOWER LIMB AND HIP

B327z00 MALIGNANT MELANOMA OF LOWER LIMB OR HIP NOS

B324100 MALIGNANT MELANOMA OF NECK

B323.00 MALIGNANT MELANOMA OF OTHER AND UNSPECIFIED PARTS OF FACE

B32y.00 MALIGNANT MELANOMA OF OTHER SPECIFIED SKIN SITE

B325400 MALIGNANT MELANOMA OF PERIANAL SKIN

B325500 MALIGNANT MELANOMA OF PERINEUM

B327300 MALIGNANT MELANOMA OF POPLITEAL FOSSA AREA

B324000 MALIGNANT MELANOMA OF SCALP

B324.00 MALIGNANT MELANOMA OF SCALP AND NECK

B324z00 MALIGNANT MELANOMA OF SCALP AND NECK NOS

B326000 MALIGNANT MELANOMA OF SHOULDER

B32..00 MALIGNANT MELANOMA OF SKIN

B32z.00 MALIGNANT MELANOMA OF SKIN NOS

B323500 MALIGNANT MELANOMA OF TEMPLE

B327100 MALIGNANT MELANOMA OF THIGH

B326500 MALIGNANT MELANOMA OF THUMB

B327800 MALIGNANT MELANOMA OF TOE

B325.00 MALIGNANT MELANOMA OF TRUNK (EXCLUDING SCROTUM)

B325z00 MALIGNANT MELANOMA OF TRUNK, EXCLUDING SCROTUM, NOS

B325600 MALIGNANT MELANOMA OF UMBILICUS

B326100 MALIGNANT MELANOMA OF UPPER ARM

B326.00 MALIGNANT MELANOMA OF UPPER LIMB AND SHOULDER

B326z00 MALIGNANT MELANOMA OF UPPER LIMB OR SHOULDER NOS

B32y000 OVERLAPPING MALIGNANT MELANOMA OF SKIN

BBEG000 [M]ACRAL LENTIGINOUS MELANOMA, MALIGNANT

BBEA.00 [M] AMELANOTIC MELANOMA

BBE4.00 [M] BALLOON CELL MELANOMA

BBE1100 [M] DESMOPLASTIC MELANOMA, MALIGNANT

BBEP.00 [M]EPITHELIOID CELL MELANOMA

BBEG.11 [M]LENTIGO MALIGNA MELANOMA

BBEM.00 [M]MALIGNANT MELANOMA IN GIANT PIGMENTED NAEVUS

BBEG.00 [M]MALIGNANT MELANOMA IN HUTCHINSON'S MELANOTIC FRECKLE

BBEC.00 [M]MALIGNANT MELANOMA IN JUNCTIONAL NAEVUS

BBE1.00 [M]MALIGNANT MELANOMA NOS

BBE1000 [M]MALIGNANT MELANOMA, REGRESSING

BBE1.11 [M]MELANOCARCINOMA

BBE1.13 [M]MELANOSARCOMA NOS

BBE1.14 [M]NAEVOCARCINOMA

BBE2.00 [M]NODULAR MELANOMA

BBEQ.00 [M]SPINDLE CELL MELANOMA NOS

BBEH.00 [M]SUPERFICIAL SPREADING MELANOMA

Byu4000 [X]MALIGNANT MELANOMA OF OTHER+UNSPECIFIED PARTS OF FACE

Byu4100 [X]MALIGNANT MELANOMA OF SKIN, UNSPECIFIED
