# Supplementary material for: Phosphodiesterase Type 5 Inhibitors and Risk of Malignant Melanoma: Matched Cohort Study Using Primary Care Data from the UK Clinical Practice Research Datalink
Source: PLoS Med. 2016 Jun 14;13(6):e1002037. doi: 10.1371/journal.pmed.1002037 (PMC4907438; doi:10.1371/journal.pmed.1002037)
Supplement: S3 Text — (DOCX) [file pmed.1002037.s005.docx]

**S3 Text: Pre-specified and approved study protocol and explanation for changes (see over)**

(PROTOCOL) Phosphodiesterase type-5 inhibitor use and the risk of incident melanoma cancer in the UK

**Lay Summary**

In 2014, Li et al published a paper reporting evidence to suggest that the use of the phosphodiesterase type-5 (PDE5) inhibitor, sildenafil, typically used to treat erectile dysfunction, increased the risk of a type of skin cancer known as malignant melanoma, in a US-based cohort of male health professionals. This was the first epidemiological evidence to suggest an association between a PDE5 inhibitor and melanoma and as yet, has not been replicated using another data source. As NHS restrictions regarding the prescription of PDE5 inhibitors have recently been relaxed, meaning the drugs are more readily available, this finding could have important public health implications. We aim to examine the association between PDE5 inhibitors and the risk of incident melanoma among men in the UK using data from CPRD.

To examine the association between PDE5 inhibitors and melanoma we will initially compare the risk of melanoma in individuals that have ever been exposed to a PDE5 inhibitor to similar individuals that have never been exposed to a PDE5 inhibitor. We will then go on to assess the effect of the cumulative numbers of PDE5 prescriptions on the risk of incident melanoma.

**Background**

Malignant melanoma is the 6^th^ most common cancer in males the UK [1]. In 2011, the age standardised incidence rate was 17.5 (95% CI: 17.1, 18.0) cases per 100,000 men [1] and rates are significantly higher in the south and south west areas of the UK [2]. It is generally accepted that the main risk factor of malignant melanoma is exposure to UV radiation from both sun exposure and tanning beds [3].

The PDE5 inhibitor, sildenafil (marketed as Viagra), is principally used in the treatment of erectile dysfunction (ED) [4]. Li et al recently reported epidemiological evidence to suggest an increased risk of melanoma due to sildenafil use in a US based cohort of health professionals, using self-reported data [5]. However, as yet, this observation has not been replicated using another data source.

Until recently, prescription of PDE5 inhibitors on the NHS was restricted to men presenting with both ED and one of a number of pre specified diagnoses, including diabetes, renal failure and severe distress [6]. On 21^st^ June 2013, Pfizer’s patent for sildenafil expired in the UK, opening the window for other manufacturers to sell generic versions at a lower cost. Due to this change in the market, generic sildenafil has recently become available on the NHS to all men presenting with ED. The increased availability of the drug on the NHS has the potential to considerably inflate demand.

As the association found by Li et al is novel, this study will aim examine the association between both sildenafil and other PDE5 inhibitors used for erectile dysfunction and the risk of incident melanoma among men in the UK CPRD.

**Objective, specific aims and rationale**

*Objective*

To assess the effect of PDE5 inhibitors on the risk malignant melanoma in men in the UK

*Aims*

1. Assess the effect of ever using a PDE5 inhibitor on the risk of malignant melanoma
2. Assess the cumulative effect of number of PDE5 inhibitor prescriptions on the risk of malignant melanoma
3. Investigate whether individual-level characteristics modify the association between PDE5 inhibitors and malignant melanoma risk

*Rationale*

There has previously been only one study in the USA that assessed this association and found PDE5 inhibitors to increase the risk of malignant melanoma; this received considerable attention, but the finding has not since been replicated. We therefore believe it will be beneficial to explore whether this association can be replicated in a separate population.

**Study Type**

Hypothesis testing with the null hypothesis that PDE5 inhibitors have no effect on the risk of malignant melanoma in men the UK.

**Study Design**

Matched cohort study using CPRD

**Linked Data**

Patient-level index of multiple deprivation data are required to assess for confounding as it is thought that a combination of socio-demographic status and geographical location can be used as a proxy for levels of sun exposure. Since this is only available for patients in the linkage scheme, we propose to use practice-level index of multiple deprivation for the remainder of patients (rescaled to be on the same scale as the patient level scores, using observations where both are available). We have used this strategy successfully in previous CPRD studies.

**Study Population**

We will identify all male patients over the age of 18 with an incident exposure to a PDE5 inhibitor from 1^st^ July 1999 to 1^st^ August 2014 inclusive. The reasoning behind the proposed study period is that the restrictions regarding the prior diagnoses required to receive a PDE5 inhibitor prescription were imposed on 1^st^ July 1999, and restrictions for generic sildenafil were lifted on 1^st^ August 2014, after Pfizer’s sildenafil patent expired on 21^st^ June 2013. Follow up will begin at the date of first PDE5 inhibitor prescription (hereafter the “index date”). Exposed patients will matched to up to four controls on age at first prescription (within 3 years in either direction), GP practice, diabetes status, and their index date will be the same as their matched case’s. All matching variables will be measured at the index date. Patients with any cancer diagnoses prior to the index date will be excluded. Individuals selected as controls may later go on to start a PDE5 inhibitor; in this situation they will be censored as a control at the time of starting a PDE5 inhibitor, and they will contribute separately as an exposed patient from that time point (with their own matched controls).

**Sample size/power calculation**

Preliminary feasibility analyses suggest that there will be approximately 179,298 men with incident sildenafil prescriptions in the study population. The prevalence of malignant melanoma among these patients is ~0.27%. With each exposed patient matched to one control, the study would have 80% power to detect an effect size of approximately 1.18, at a significance level of 5%; our higher intended matching ratio will increase the power further. This shows that our study will be highly powered to detect an effect size similar to that observed by Li et al.

**Exposures and outcome**

*Exposure*

- Primary exposure – Ever received a PDE5 inhibitor prescription (time-updated)

An incident PDE5 inhibitor prescription will be identified by an appropriate therapy code in the patient’s therapy records (relevant code list in appendix 1). To be considered incident, the first prescription must be at least 12 months after initial registration into CPRD as prescriptions within this period could be continuation of a prescriptions from a prior GP, of unknown duration. All matched controls must also have one year of post registration follow up prior to index date.

- Secondary exposure – Cumulative number of PDE5 inhibitor prescriptions (time-updated)

All subsequent prescriptions will be identified and the cumulative number of prescriptions will be categorised into the following groups: 1, 2-4, 5-10, 10-20 and 20+.

Number of prescriptions prescribed to patients will be used as a secondary exposure instead of cumulative duration of prescription because PDE5 inhibitors are prescribed for a patients to take as required. We are unable to judge the sexual activity of patients, hence any estimations made in an attempt to identify the length of time until a patient runs out of tablets will be unreliable. As a further analysis we will use the cumulative number of tablets prescribed to a patient as an exposure.

*Outcome*

The primary outcome is incident malignant melanoma, identified by an appropriate Read code in the patients’ medical record (relevant code list in appendix 2).

**Statistical/analysis plan**

Within the cohort, a patient’s observation time will begin at the index date and will end when one of the following occur: incident malignant melanoma, at diagnosis of a cancer other than malignant melanoma, death, transfer out of CPRD network, prescription of a PDE5 inhibitor (for unexposed controls), or end of follow up. The following analyses will be carried out using incident malignant melanoma as the primary outcome.

*Descriptive analyses*

Prior to exploring the relationship between PDE5 inhibitors and malignant melanoma, we will firstly describe the distribution of exposed and unexposed within the following variables: age, ever prescribed a PDE5 inhibitor, GP practice region (North East, North West, Yorkshire & the Humber, East Midlands, West Midlands, East of England, South West, South Central, London, South East Coast, Northern Ireland, Scotland, Wales), diabetes status, smoking status (non-smoker, current smoker, ex-smoker), BMI (underweight, normal, overweight/obese), alcohol status (non-drinker, current drinker, ex-drinker), index of multiple deprivation score.

*Ever exposed vs. never exposed to a PDE5 inhibitor*

Crude incidence ratios will firstly be calculated. The main exposure variable will then be included in an unadjusted and adjusted Cox regression model (see “Confounding” below), stratified by matched set to account for the matching. The primary outcome will be a diagnosis of incident melanoma and patients will be censored at diagnosis of any other cancer, death, transfer out of the CPRD network, or on 21^st^ June 2013 (end of follow up). Hazard ratios and 95% confidence intervals will be estimated, comparing the rate of incident melanoma between patients who have ever been prescribed a PDE5 inhibitor and patients that have never been prescribed a PDE5 inhibitor. We will also calculate changes in absolute risk of melanoma by obtaining predictions from a Poisson model with the same covariates as our main Cox model, plus time since index date (time-updated).

*Cumulative duration of exposure*

Unadjusted and adjusted Cox regression models will then be re-run using a time updated variable capturing the cumulative number of PDE5 inhibitor prescriptions in place of the ever exposed variable. Hazard ratios and 95% confidence intervals will again be estimated.

*Confounding*

Age, GP practice, diabetes status and calendar time are matching variables. Our models will also be adjusted for the following potential confounders evaluated at beginning of follow up:

- Smoking status (never-smoker, current smoker, ex-smoker)
- BMI (underweight, normal, overweight, obese)
- Alcohol status (never-drinker, current drinker, ex-drinker)
- Index of multiple deprivation score

*Interaction*

We will fit interaction terms to explore possible effect modification between exposure to a PDE5 inhibitor and age, smoking status and index of multiple deprivation score.

*Sensitivity analysis*

As a sensitivity analysis we will exclude the first 12 months of follow up after index date in both exposed and unexposed patients. This is to decrease the chance of reverse causality, whereby undiagnosed melanoma cancers cause ED and hence the need for a PDE5 inhibitor prescription.

We will also conduct an analysis restricted to patients diagnosed with diabetes as previous restrictions mean all patients diagnosed with diabetes were definitely eligible for a PDE5 prescription.

**Limitations**

*Missing data*

Although there is known missingness in the smoking and alcohol status and BMI variables, this is minimal. Initial feasibility analyses using a CPRD random sample show that 2.3% and 1% of patients prescribed a PDE5 inhibitor have missing data for alcohol status and smoking status respectively. We plan to conduct a complete case analysis, which relies on the assumption that the probability of these data being missing is independent of melanoma risk, conditional on covariates [7]; given the small amount of missing data, any violation of the assumption is unlikely to importantly affect the results.

*Lack of data on UV Exposure*

UV exposure is a strong risk factor for malignant melanoma, though it is unclear as to whether UV exposure is likely to be associated with PDE5 inhibitor use and therefore a potential confounder. As data on patient level UV exposure are unavailable, we will match on GP practice as we believe patients from the same practice will have similar sun exposures whilst at home due to geographical location, though a limitation is that it will be impossible for us to take account of amount of time spent outdoors. We will also adjust for index of multiple deprivation score as we believe this will to some extent act as a proxy for amount of travel abroad.

*Sale of PDE5 inhibitors without a prescription*

There is a high volume of PDE5 inhibitors, especially sildenafil (marked as Viagra), sold illegally. It is therefore possible that patients could obtain medication without a prescription resulting in exposure status misclassification. If this is the case, it would result in an underestimation of the true effect size.

**Plans for disseminating and communicating results**

The study findings will be submitted for publication in peer-reviewed scientific journals, and will be presented at appropriate conferences and other meetings. We will engage fully in opportunities to communicate our results to the general public, including via the media.

Protocol references

1. *Skin Cancer Incidence Statistics*. Cancer Research UK - Cancer Statistics 2014 [cited 2015 10/03/2015].

2. Quinn, M., H. Wood, and N. Cooper, *Cancer Atlas of the United Kingdom and Ireland 1991-2000*, in *Studies of Medical Population Subjects*, O.o.N. Statistics, Editor. 2005: London.

3. Cogliano, V.J., et al., *Preventable exposures associated with human cancers.* J Natl Cancer Inst, 2011. **103**(24): p. 1827-39.

4. *British National Formulary* Vol. 63. 2012, London: BMJ Group.

5. Li, W.Q., et al., *Sildenafil use and increased risk of incident melanoma in US men: a prospective cohort study.* JAMA Intern Med, 2014. **174**(6): p. 964-70.

6. *Proposed changes to NHS availability of erectile dysfunction treatments - changing prescribing restrictions for generic sildenafil*, D.o. Health, Editor. 2014, gov.uk: London.

7. White, I.R., Carlin, J.B., *Bias and efficiency of multiple imputation compared with complete-case analysis for missing covariate values*, Statistics in Medicine, 2010. 29 2920--2931.

**CHANGES FROM THE PROTOCOL AND EXPLANATION**

We added an extra exclusion criteria: men with no consultation in the year prior to the index date were excluded from the study. This is because we initially discovered a large discrepancy in the proportion of non-consulting patients between the exposed and unexposed groups, with the unexposed much more likely to be non-consulters, implying a potential for substantial differences in ascertainment of outcomes, and therefore bias. All analyses were otherwise carried out as pre-specified, but we conducted some further analyses to assess causality and exclude bias, as follows:

1. We added an extra secondary analysis to investigate changes in the association between PDE5 use and melanoma by time since first prescription date. This was to further explore whether patterns of effects were consistent with causality.

2. We investigated interaction by practice region, as well as by the pre-specified potential effect modifiers. This was because of an apparent important role for sun exposure; the climate in the southern UK is generally warmer with more hours of sunshine than in the northern part of the country.

3. We added three negative control outcomes (basal cell carcinoma, solar keratosis, colorectal cancer) as a way of excluding bias. No causal effects of PDE5 inhibitor use were postulated for these outcomes. The specific outcomes were chosen to incorporate one non-melanoma skin malignancy, one non-malignant skin condition, and one condition unrelated to skin.

4. We conducted a post-hoc analysis to relate PDE5 exposure to prior solar keratosis, in order to assess possible confounding by sun exposure.
